# Weight Loss Predicts Progression of Mild Cognitive Impairment to Alzheimer’s Disease

**DOI:** 10.1371/journal.pone.0151710

**Published:** 2016-03-18

**Authors:** Ilaria Cova, Francesca Clerici, Annalia Rossi, Valentina Cucumo, Roberta Ghiretti, Laura Maggiore, Simone Pomati, Daniela Galimberti, Elio Scarpini, Claudio Mariani, Barbara Caracciolo

**Affiliations:** 1 Center for Research and Treatment on Cognitive Dysfunctions, Biomedical and Clinical Sciences Department, “Luigi Sacco” Hospital, University of Milan, Milan, Italy; 2 Neurology Unit, Department of Pathophysiology and Transplantation, University of Milan, Fondazione Cà Granda, IRCCS Ospedale Policlinico, Milan, Italy; 3 Aging Research Center, Department of Neurobiology, Health Care Sciences and Society, Karolinska Institutet, and Stockholm Gerontology Research Center, Stockholm, Sweden; Biomedical Research Foundation, UNITED STATES

## Abstract

**Background:**

Weight loss is common in people with Alzheimer’s disease (AD) and it could be a marker of impending AD in Mild Cognitive Impairment (MCI) and improve prognostic accuracy, if accelerated progression to AD would be shown.

**Aims:**

To assess weight loss as a predictor of dementia and AD in MCI.

**Methods:**

One hundred twenty-five subjects with MCI (age 73.8 ± 7.1 years) were followed for an average of 4 years. Two weight measurements were carried out at a minimum time interval of one year. Dementia was defined according to DSM-IV criteria and AD according to NINCDS-ADRDA criteria. Weight loss was defined as a ≥4% decrease in baseline weight.

**Results:**

Fifty-three (42.4%) MCI progressed to dementia, which was of the AD-type in half of the cases. Weight loss was associated with a 3.4-fold increased risk of dementia (95% CI = 1.5–6.9) and a 3.2-fold increased risk of AD (95% CI = 1.4–8.3). In terms of years lived without disease, weight loss was associated to a 2.3 and 2.5 years earlier onset of dementia and AD.

**Conclusions:**

Accelerated progression towards dementia and AD is expected when weight loss is observed in MCI patients. Weight should be closely monitored in elderly with mild cognitive impairment.

## Introduction

Dementia, particularly Alzheimer’s disease (AD), has become a major public health concern due to population aging. Dementia can be preceded by a condition known as mild cognitive impairment (MCI), a term that covers a broad spectrum of cognitive impairment syndromes [1]. MCI has an annual progression rate to dementia and to AD of 9,6% and 8,1%, respectively in specialist clinical settings, and a rate of 4.9% and 6.8%, in community studies [2]. During the last decade, considerable effort has been devoted to the identification of factors that can impact on the progression of MCI to dementia [3], but the picture is far from being exhaustive. Weight loss is common in people with Alzheimer’s disease (AD) [4] and a "slow progressive decrease in body weight" was already reported by Alois Alzheimer. Therefore, weight loss could be used as a marker of impending AD in people with MCI, however, evidence on weight loss as a predictor of progression to dementia and AD in MCI needs to be replicated [5]. If demonstrated, such an association may help understanding the mechanisms underlying the neurodegenerative cascade leading to AD.

The aim of this study was to investigate weight change as a predictor of progression of MCI to dementia and AD. Specifically we aimed to investigate whether weight loss increases the risk of dementia and AD in patients with MCI recruited from a memory clinic.

## Materials and Methods

### Study Population

We enrolled outpatients from the Center for Research and Treatment of Cognitive Dysfunctions, Sacco Hospital, University of Milan, consecutively admitted from January 2001 to September 2012. One-hundred-twenty-five subjects fulfilled inclusion criteria for a clinical diagnosis of MCI.

Yearly check-ups were performed as part of the clinical routine. Follow-up ended at the diagnosis of dementia [6], death of subjects or at the end date for dementia surveillance (May 2014). The Sacco hospital ethics committee approved the study protocol and informed written consent from all subjects was obtained by the treating physicians, after neuropsychological assessment of the patients' capacity to provide a consent (no patients wth dementia have been included in this study).

### Baseline assessment

At baseline, all study participants underwent a clinical evaluation following a standardized diagnostic protocol. Collected data included demographic characteristics, medical history, actual and previous pharmacological treatments, clinical and neurological examination, standard laboratory blood tests and neuroimaging (MRI or CT scan). Each subject underwent an extensive neuropsychological examination.

### Neuropsychological Assessment

The neuropsychological battery included the Mini Mental State Examination (MMSE) [7] and the Clinical Dementia Rating (CDR) [8] as tests of general cognition, and specific tests were administered to assess the following cognitive domains: verbal memory (Story Recall Test, immediate and delayed recall of Rey’s Auditory Verbal Learning Test, recall of Rey-Osterrieth Complex Figure Test), language (Letter and Category Fluency Test), attention and executive functions (Weigl’s Test, Frontal Assessement Battery, Trail Making Test), and visuo-spatial ability (Copy of Rey-Osterrieth Complex Figure Test). Cut-off scores (≥ 95% of the lower tolerance limit of the normal population distribution) were based on previous research on Italian samples[9,10]. Functional status was assessed with the Activities of Daily Living (ADL) [11] and the Instrumental Activities of Daily living (IADL) scales [12]. Presence and severity of psychiatric symptoms were evaluated by the Neuropsychiatric Inventory [13]. Depression was assessed using the 30-item version of the Geriatric Depression Scale [14].

### MCI diagnosis

MCI was diagnosed according to standard criteria [15] and operationalized as follows: (1) subjective cognitive complaint as reported by subjects and corroborated by an informant; (2) objective cognitive impairment, defined as scoring below 1.5 standard deviation from the mean (according to age- and education-specific norms) on at least one task of a previously described neuropsychological assessment; (3) essentially preserved daily functioning defined as no impairment in both basic and instrumental activities of daily living, and (4) absence of dementia defined according to DSM-IV criteria [6].

### Outcome

The outcome was a diagnosis of dementia that required evidence of cognitive decline on the neuropsychological test battery and of impairment on social or occupational functioning, as specified by the DSM-IV criteria [6]. Subjects were tested by specialized clinical neuropsychologists. Diagnosis of dementia and dementia subtype was established after collective decision by at least a neurologist and a neuropsychologist. AD was diagnosed according to NINCDS-ADRDA criteria [16], Lewy body dementia according to McKeith criteria [17], Frontotemporal dementia according to Lund and Manchester criteria [18] and vascular dementia was defined according to NINDS-AIREN criteria [19].

Three patients who refused the follow-up were diagnosed by phone interview applying a validated informant-based Clinical Dementia Rating scale score [20].

### Predictors

Participant height and body weight were recorded at each visit. BMI (kg/m2) was calculated from height and weight at baseline and categorized as obese (> = 30 kg/m2), overweight (25–29,9 kg/m2), normal weight (20–24,9 kg/m2), or underweight (< 20 kg/m2). Weight change was calculated as the difference between a subject’s weight at the first visit and the last follow-up. Based on previous research [21–23] we used a 4% WC cut-off point to identify three groups of MCI according to weigh change over time: MCI with ≥ 4% weight loss, MCI with ≥ 4% weight gain, or stable weight MCI (< 4% weight gain or loss).

### Covariates

Potential confounders included personal data, such as: age at first visit, sex, years of education, MMSE, MCI subtype, smoking habits (previous smoker, actual smoker, no smoker). Somatic comorbidity was quantified using the Modified Cumulative Illness Rating Scale (CIRS) [24].

The modified CIRS includes 14 categories assessing the impairment of each organ system, with a score ranging from 0 to 4. A score of “0” means that the system considered has no clinical dysfunction or has a clinically irrelevant dysfunction; a score of “1” means that the system considered has a mild clinical dysfunction, or has had a relevant dysfunction for which no treatment is required; a score of “2” means that the subject has a moderate clinical condition/disability that requires a first-line treatment; a score of “3” means that the subject has a severe condition and/or disability and/or a chronic condition requiring a complex treatment; a score of “4” means that the subject has a very severe condition and/or needs an urgent treatment due to an organ failure and/or severe functional disability. The total score was calculated adding the scores from each of the 14 individual system scores. The “CIRS comorbidity index”, based on the sum of CIRS items with scores ≥ 2 (indicating moderate disability or morbidity and/or requires first line therapy) was also calculated. Further covariates were: diabetes mellitus (fasting venous plasma glucose > = 126 mg/dl and/or treatment for diabetes mellitus), depression (clinical depression with/without treatment and/or GDS score ≥ 11) and cerebrovascular disease (history of previous transient ischemic attack or stroke). We used the Age-Related White Matter Changes Scale (ARWMC) [25,26] to rate subcortical cerebrovascular disease. The ARWMC scale was dichotomized into the absence of WMLs (i.e. ARWMC score = 0 in all areas) versus the presence of them (all other cases).

APOE genotyping, was available for a subgroup of subjects (102 subjects) and was dichotomized in being a carrier of at least one allele ε4 and carrying no ε4 allele.

### Data analysis

Subjects were considered at risk of dementia until one of the following outcomes occurred: 1) incident dementia; 2) death; 3) end of the study. Subjects’ characteristics at baseline were compared by outcome using the ANOVA test for continuous variables and Pearson’s χ^2^ test for categorical variables.

The relative risk of progression to dementia in relation to weight change was assessed by means of Cox regression models. Unadjusted and partially adjusted (for sociodemographic variables such as age, gender and education) models were carried out. The fully adjusted model included smoking habits, diabetes mellitus, depression, cerebrovascular disease, cerebral white matter lesions on brain imaging, and comorbidity. For 102 subjects it was possible to further adjust also for APOE genotype. These variables were included as they all could have confounded or modified the longitudinal relationship between body weight and dementia.

The same models and analytic strategy were repeated using AD rather than dementia as an outcome. Cumulative hazard curves for dementia and AD were derived from the fully adjusted models for dementia and AD.

All analyses were performed using SPSS 21 with an α level of p <0.05.

## Results

Out of 125 subjects, 53 (42.4%) progressed to dementia with AD accounting for 55% of all cases (n = 29), 3 subjects (6%) progressed to Lewy body dementia, 5 (9%) to vascular dementia and 8 (15%) to unspecified dementia.

Median time from baseline to dementia occurrence was 4.01 ± 2.47 years.

Table 1 shows demographic and clinical characteristics at baseline of the whole sample (mean age 73.8 ± 7.08 years, mean education 7.82 ± 4.18 years, mean score in MMSE 25.78 ± 2.63) and by cognitive outcome at follow-up.

**Table 1:** Baseline characteristics and comorbidities in the whole sample, in stable MCI at follow up and in MCI progressed to dementia.

Demographic and clinical characteristics at baseline	MCI at baseline N = 125	MCI at follow-up N = 72	Dementia at follow-up N = 53	p
**Age**				
<75 years, n (%)	66 (52.8)	42 (58.3)	24 (45.3)	n.s.
>75 years, n (%)	59 (47.2)	30 (41.7)	29 (54.7)	
**Sex**				
Men, n (%)	57 (45.6)	35 (48.6)	22 (41.5)	n.s.
Women, n (%)	68 (54.4)	37 (51.4)	31 (58.5)	
**Education**				
< 9 years, n (%)	89 (71.2)	53 (73.6)	36 (67.9)	n.s.
≥ 9 years, n (%)	36 (28.2)	19 (26.4)	17 (32.1)	
**Cognitive status**				
Low (MMSE < 26), n (%)	47 (37.6)	21 (29.2)	26 (49.1)	0.023
High (MMSE ≥ 26), n (%)	78 (62.4)	51 (70.8)	27 (50.9)	
**Depression**				
No, n(%)	49 (39.2)	29 (40.3)	20 (37.7)	n.s.
Yes, n (%)	76 (60.8)	43 (59.7)	33 (62.3)	
**MCI subtypes**				
Amnestic single domain, n (%)	32 (25.6)	20 (27.8)	12 (22.6)	n.s.
Non amnestic single domain, n (%)	19 (15.2)	14 (19.4)	5 (9.4)	
Multiple domain, n (%)	74 (59.2)	38 (52.8)	36 (67.9)	
**APOE4 genotype** *				
No allele ε 4, n (%)	70 (68.6)	44 (73.3)	26 (71.9)	n.s.
At least one allele ε 4, n (%)	32 (14.2)	16 (26.7)	16 (38.1)	
**Body Mass Index, kg/m**^**2**^				
Underweight (<20), n (%)	8 (3.5)	3 (4.3)	5 (9.8)	n.s.
Normal weight (20–24.9), n (%)	46 (36.8)	20 (37.1)	26 (39.2)	
Overweight (25–29,9), n (%)	50 (40)	30 (42.9)	20 (39.2)	
Obese (>30), n (%)	17 (13.6)	11 (15.7)	6 (11.8)	
**Weight change %**				
Weight loss ≥ 4%, n (%)	45 (36)	17 (23.6)	28 (52.8)	0.001
Weight loss or gain < 4%, n (%)	65 (52)	42 (58.3)	23 (43.4)	
**Smoking**				
No smokers, n (%)	66 (52.8)	40 (55.6)	26 (49.1)	n.s
Ex smokers, n (%)	43 (34.4)	25 (34.7)	18 (34)	
Smokers, n (%)	16 (12.5)	7 (9.7)	9 (17)	
**Diabetes**				
No, n (%)	100 (80)	58 (80.6)	42 (79.2)	n.s
Yes, n (%)	25 (20)	14 (19.4)	11 (20.8)	
**Cerebrovascular diseases**				
No, n (%)	109 (87.2)	59 (81.9)	50 (94.3)	0.04
Yes, n (%)	16 (12.8)	13 (18.1)	3 (5.7)	
ARWMC**				
score = 0, n (%)	28 (22.4)	19 (27.1)	9 (17.3)	n.s.
score≥1, n (%)	94 (75.2)	51 (72.9)	43 (82.7)	
**CIRS Upper gastrointestinal**				
score = 0	109 (87.2)	62 (86.1)	47 (88.7)	n.s.
score = 1	5 (4)	1 (1.4)	4 (7.5)	
score = 2	11 (8.8)	9 (12.5)	2 (3.8)	
**CIRS Lower gastrointestinal**				
score = 0	114 (91.2)	66 (91.7)	48 (90.6)	n.s.
score = 1	3 (2.4)	1 (1.4)	2 (3.8)	
score = 2	8 (6.4)	5 (6.9)	3 (5.7)	
**CIRS Hepatic**				
score = 0	121 (96.8)	69 (95.8)	52 (98.1)	n.s.
score = 1	1 (0.8)	0 (0)	1 (1.9)	
score = 2	3 (2.4)	3 (4.2)	0 (0)	
**CIRS Musculoskeletal**				
score = 0	91 (72.8)	47 (65.3)	44 (83.0)	n.s.
score = 1	8 (6.4)	6 (8.3)	2 (3.8)	
score = 2	23 (18.4)	17 (23.6)	6 (11.3)	
score = 3	3 (2.4)	2 (2.8)	1 (1.9)	
**CIRS Endocrine/ metabolic**				
score = 0	32 (25.6)	17 (23,6)	15 (28.3)	n.s.
score = 1	35 (28)	19 (26.4)	16 (30.2)	
score = 2	54 (43.2)	34 (47.2)	20 (37.7)	
score = 3	4 (3.2)	2 (2.8)	2 (3.8)	

* APOE available in 102 subjects

** ARWMC: Age Related White Matter Changes

The two groups (stable MCI and MCI who progressed to dementia) did not differ at baseline with respect to the main features considered, with the exception of MMSE scores: as expected, MCI who progressed to dementia had lower MMSE scores (Pearson's X2 test p = 0.013).

**Fig 1:**
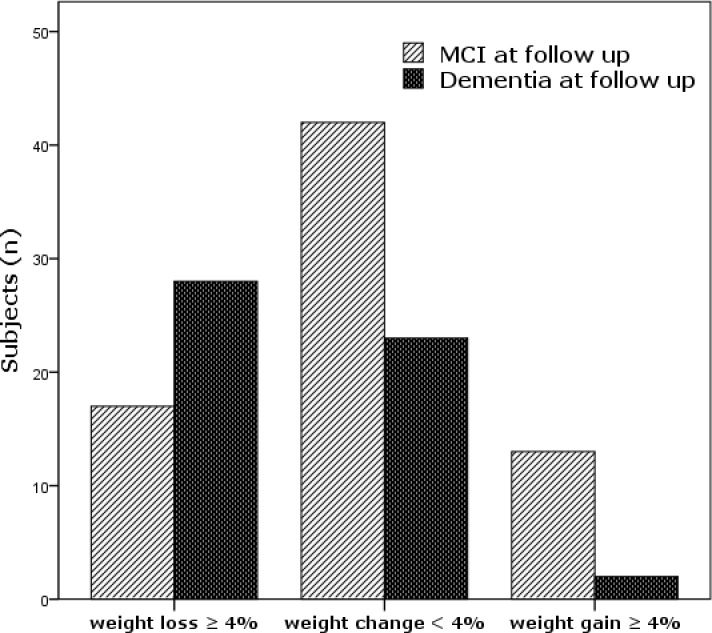
Progression to dementia of MCI subjects divided into three categories of weight change.

With regard to anthropometric measurements, the second and last weight assessment was carried out after 2.9 years ± 2.1 years from the baseline assessment. While there was no difference in baseline BMI between MCIs who progressed and those who did not progress, Incident dementia was higher in MCIs who lost weight during follow-up (62.2%) compared to subjects with no weight change (35.4%) or weight gain (13.3%) (Pearson's X2 test p = 0.001) (Fig 1).

MCI subjects who progressed to dementia did not differ from the rest of the population in terms of baseline VRFs and ARWMC scale scores (Table 1), with the exception of cerebrovascular disease, which was more prevalent among subjects who did not progress to dementia (Pearson’s χ2 p = 0.04).

Similarly, as shown in Table 1, MCI subjects who progressed to dementia did not differ from the overall MCI sample in terms of comorbidity.

**Fig 2:**
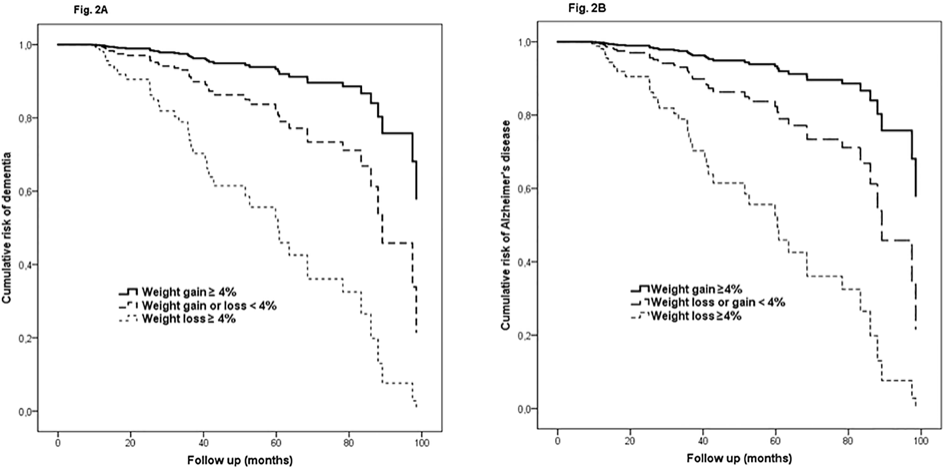
Cumulative risk curves of the effects of body weight change on progression from mild cognitive impairment (MCI) to dementia (A) and Alzheimer’s disease (B). The figure derived from a model adjusted for sex, age, education, Mini-Mental State Examination score, MCI subtypes, APOE genotype, smoking habit, depression, diabetes, cerebrovascular disease, ARWMC score, comorbidity total score.

Cox analysis showed that MCI subjects who lost ≥ 4% of their baseline weight had a significantly increased risk of progressing from MCI to dementia (HR 3.2; 95% CI 1.5 to 6.9) (Table 2 and Fig 2A).

**Table 2:** Relative risk of progression from MCI to dementia and Alzheimer's disease in relation to weight change: Cox regression model adjusted for the potential confounding variables in 102 subjects.

	Dementia			AD		
Weight change	Model 1^b^	Model 2^b^	Model 3^c^	Model 1^a^	Model 2^b^	Model 3 ^c^
	HR (95% CI)	HR (95% CI)	HR (95% CI)	HR (95% CI)	HR (95% CI)	HR (95% CI)
No weight change	1	1	1	1	1	1
Weight gain	0.4 (0.1–3.3)	0.4 (0.9–1.6)	0.3 (0.4–2.9)	0.4 (0.1–3.3)	0.4 (0.1–1.7)	0.3 (0.3–3.5)
Weight loss	1.9 (1.1–1.6)*	2.7 (1.4–5.6)*	3.2 (1.5–6.9) *	1.8 (1.1–1.6)*	2.3 (1.3–1.7)*	3.4 (1.4–8.3) *

^a^ Adjusted for age, sex, education

^b^ Adjusted for sex, age, education, Mini-Mental State Examination score, MCI subtypes, smoking habit, depression, diabetes, cerebrovascular disease, ARWMC score, comorbidity total score.

^c^ Adjusted for sex, age, education, Mini-Mental State Examination score, MCI subtypes, APOE genotype, smoking habit, depression, diabetes, cerebrovascular disease, ARWMC score, comorbidity total score (performed on 102 subjects).

* Significant with p<0.05.

Other factors associated with a significant increased risk of progression from MCI to dementia were multi-domain MCI (HR, 3.4; 95% CI 1.0 to 11.4), vascular lesions at neuroimaging (HR 3.0; 95% CI 1.0 to 8.9) and the presence of at least one APOE-ε 4 allele (HR, 2.4; 95% CI 1.0 to 6.0). As expected, higher baseline MMSE was associated with a reduced risk of progression to dementia (HR, 0.7; 95% CI 0.6–0.8).

Similar results were observed with AD as the outcome. A weight loss ≥ 4% was associated with a significantly increased risk of progression from MCI to AD (HR, 3.4; 1.4 to 8.3 95% CI) (Table 2 and Fig 2B).

Regarding other covariates, as expected, lower education was associated with an increased risk of progression to AD (HR, 1.3; 95% CI 1.1–1.5), while higher baseline MMSE scores were associated with a reduced risk (HR, 0.7; 95% CI 0.6–0.8)

Cumulative risk curves derived from Cox models showed an average onset time for dementia of 5 years in MCI who lost weight compared to 7.4 years in MCI with stable weight (Fig 2A). Similarly, average onset time for AD was 5.6 years in MCI who lost weight compared to 8.1 years in stable weight MCI (Fig 2B).

## Discussion

In this clinical sample, MCI who lost ≥ 4% of their body weight during follow-up had a 3.4-fold increased risk of dementia and a 3.2-fold increased risk of AD. On average, weight loss was associated with a 2.3 and 2.5 years earlier onset of dementia and AD. This is in agreement with previous findings which showed a 30–40% weight loss in mild to moderate AD patients [22,27] and with studies suggesting weight loss begins years before the diagnosis of AD [28,29]. It is also consistent with results from a population-based study which found that amnestic MCI who lose weight undergo faster functional decline after one year [21]. In addition to weight change, we found other factors that influenced the progression from MCI to dementia and AD: higher baseline MMSE scores were associated with a reduced risk [30] while the presence of vascular lesions, as calculated by the ARWMC scale [26], was associated with an increased risk.

Our study design does not allow to determine the direction of the association between weight loss and the dementing process. It remains unclear whether weight loss is only a manifestation of impending dementia and AD or a “true” risk factor for the progression of MCI.

Weight loss in the elderly is likely to be associated with deficiency in important micronutrients, such as vitamins and essential fatty acids, which can give rise to secondary oxidative stress and tissue’s damage [4]. Furthermore, dietary restrictions have been associated with a decline in vigilance, slower reaction times and worsening in immediate memory [31].

Losing weight could be a consequence of progressive cognitive deterioration, which involves ability to purchase food, as well as that of cooking meals [32] or the tendency to forget to eat. Cognitive decline and maybe concomitant depressed mood [33] could therefore be associated with a reduced food intake leading to involuntary weight loss. It has been found that apathy has a strong association with loss of weight in nursing home residents with dementia [34]. Along with progressive cognitive impairment in elderly with MCI, adequate nutritional status is also dependent on caregivers’ support. Alternatively, weight loss could be a clinical feature of the dementing process. A decreased olfaction and hence decreased appetite has been described in MCI who progress to dementia [35]. Changes in the hormonal regulation of energy metabolism can also contribute to alteration in body weight and to cognitive decline. The hypothalamus, particularly the CA1 region, a pathognomonic area affected by early atrophy in the course of AD pathology, is involved in energy homeostasis through control of hunger and satiety [36]. Other organs regulate production of hormones directly interacting with the hypothalamus in the regulation of energy expenditure, such as adipose tissue which produces leptin, responsible of hyperphagic responses [37]. Administration of leptin in the hippocampus in mice improves the processing of the memory and also increases the expression of NMDA receptors [38]. A case-control study showed that AD patients with a low BMI (<20 kg / m^2^) had lower concentrations of leptin compared to cognitively normal people of normal weight (BMI 20–25 kg / m^2^), and a positive association between BMI and leptin was found in the whole sample [39]. Dysregulation in the activity of, and sensitivity to, leptin and other peptides regulating the appetite is also described in the context of a phenomenon called “anorexia of aging”. It is a borderline state between physiological and pathological reduction of appetite frequently observed in the elderly due to a reduction of central feeding drive. Elderly with dementia, particularly of the Alzheimer’s type, had a nearly two-fold increased risk of anorexia compared with those who were dementia-free [40].

Another important factor in cognitive decline concomitant with weight loss could be played by an imperfect functioning of the mitochondria [41]. Conformational changes, alteration in electric potential, mobility and oxidative stress response are described in mitochondria of rat neurons during contemporary exposure to beta amyloid and phosphorylated tau [42]. Mitochondrial dysfunction could justify typical reduction of cerebral metabolism in AD, in particular of the temporal-parietal cortex [41]. Mitochondria supply brain cells of about 90% of the total energy request [43]. An uncoupling of the system of transport of the electron transport system, which is a distinctive characteristic of the brown adipose tissue, leads to a reduced production of ATP and to the production of heat. This mechanism represents a cytoprotective strategy used in the aging processes to reduce the production of free radicals [44]. However, uncoupling that occurs in altered mitochondria can increase the production of free radicals, which induces cell damage and increases the permeability of the mitochondrial membrane to H + ions, supporting the process of uncoupling in a vicious circle [45]. An increase in mitochondrial oxidative enzyme in the muscle tissue of AD compared to controls was found in 1991, supporting hypermetabolic hypothesis [46]. Finally hypermetabolism seems to be powered by neuro-inflammation. The amyloid deposits activate astrocytes and microglia in the production of cytokines, responsibles for a systemic inflammatory response and for suppressing feeding by taking effect on the glucose-sensitive neurons in the hypothalamic centers of satiety and hunger [47–49]. Chronic inflammation may mediate also muscle loss, accelerating an age-related process (sarcopenia); a reduction of muscle mass could be also due to a decreased physical activity. [50]

Major strengths of this study are: 1) the use of a strong endpoint, such as progression to dementia, instead of other surrogate endpoints, such as variations in scores of cognitive or functional scales. Dementia was chosen as the primary outcome of this study because of its relevance in terms of both public and individual health. Interestingly, in stratified analysis by dementia subtype, weight loss was associated also with AD. The latter finding, if replicated, could help to understand the etiology of AD; 2) the clinical setting in which the study took place (memory clinic) allows to generalize the results to populations of MCI attending memory clinics; 3) detailed baseline collection of several potential confounding factors (particularly with regard to metabolic disorders and depression), which allowed adjustment for variables which are often not available in population-based studies.

The findings reported in this study should be considered in the context of its limitations. These include: 1) heterogeneity of the construct of MCI. MCI is a risk condition characterized by a high variability (especially in terms of rate of progression to dementia) according to the diagnostic criteria used and the setting in which it is studied [1]. Nearly 42.4% MCI progressed to dementia over a period of almost four years, resulting in a progression rate of over 10% per year. This rate is slightly higher than that reported in population-based studies [2] but is consistent with what was reported in previous clinical studies, confirming that memory clinics represent selected subpopulations of people with an increased risk of progression to dementia as compared to those included in population-based studies. 2) the lack of neuropsychological assessment of apathy, which could be linked to weight loss as previously reported [34] And, 3) limitations in the measure of body weight, which does not distinguish between the various components of body mass. So, when considering a weight loss, this may correspond to a reduction in muscle mass, fat mass, or both. It may therefore be interesting to further differentiate the role of body composition using more sensitive measures, designed to detect changes in different parts of the body mass, since muscle tissue and adipose tissue organs are metabolically different and have different implications for the risk of dementia.

## Conclusions

Our study provides clinically relevant information for health care professionals working with elderly patients with MCI. Clinicians should be aware of the importance of weight loss as a predictor of the progression of MCI to dementia and consider the opportunity of a multidimensional approach in follow-up visits, including anthropometric measures and serum controls to detect malnutrition.

Weight loss in elderly with mild cognitive impairment should be closely monitored.
